# Atraumatic Lateral Tibial Plateau Periprosthetic Insufficiency Fracture after Primary Total Knee Arthroplasty: A Case Report

**DOI:** 10.3390/reports7020044

**Published:** 2024-06-04

**Authors:** Ahmed M. Abdelaal, Ahmed A. Khalifa

**Affiliations:** 1 Orthopaedic and Traumatology Department, Assiut University Hospital, Assiut 83523, Egypt; 2 Hospital for Advanced Orthopaedics, Assiut 83523, Egypt; 3 Orthopedic Department, Qena Faculty of Medicine and University Hospital, South Valley University, Qena 83523, Egypt

**Keywords:** periprosthetic fracture, total knee arthroplasty, atraumatic fracture, proximal tibia

## Abstract

Tibial Periprosthetic fractures (PPF) after primary total knee arthroplasty (TKA) are uncommon and mainly occur after trauma. Various management options have been proposed; however, the decision mainly relies on the location of the fracture and the tibial baseplate stability and ranges between conservative (non-operative), fracture fixation, and revision TKA. We report a case of a 79-year-old female patient who presented with atraumatic lateral tibial plateau PPF (Felix type ⅠB) with a loose tibial implant after three weeks of having left primary TKA. The patient was treated successfully by revising the tibial component using a stemmed tibial baseplate and reconstructing the tibial bone defect using two metal wedges. The radiological, functional, and PROM outcomes were satisfactory and accepted both early on (eight weeks) and at the last follow-up (six months). Atraumatic insufficiency Felix type ⅠB PPF of the lateral tibial plateau after primary TKA is uncommon. Reconstructing the tibial bone defect, revising the tibial component, and adding a stem to offload the tibial plateau are the treatments of choice that lead to acceptable outcomes.

## 1. Introduction

Total knee arthroplasty is a safe procedure with favorable outcomes and survival rates reaching up to 94.8% and 92.7% at 10 and 15 years of follow-up, respectively [1,2,3].

Recently, revision for periprosthetic fractures (PPF) after TKA has become a genuine concern to orthopedic surgeons, representing the fourth most common cause for revision TKA (3.6%) after aseptic loosening (24.7%), periprosthetic joint infection (23.7%), and patellofemoral problems (9.1%), as reported by the Australian Orthopaedic Association National Joint Replacement Registry (AOANJRR) [1,3].

PPFs after TKA can occur early postoperatively or late [1,4,5], and is mainly due to trauma; however, in osteoporotic bones, these could be atraumatic or “insufficiency fractures” [6,7,8,9].

Proximal tibial PPFs after TKA are considered rare, occurring at an incidence between 0.4 and 1.7%, according to the AOANJRR [3]. These fractures are frequently reported as intraoperative complications during tibial preparation, while positioning a stemmed tibial component, or even while removing the tibial trial [5,10,11]. However, obesity, osteoporosis, and chronic steroid use have been reported as predisposing factors for traumatic and atraumatic (insufficiency) tibial PPFs [5,9,12,13,14]. 

Felix et al. (Mayo Classification system) is the most commonly used system to classify tibial PPFs after TKA [15]. It consists of four types based on the fracture’s anatomical location: type Ⅰ is a fracture near the tibial baseplate (the tibial plateau), type Ⅱ is a fracture around the tibial component stem (keel), type Ⅲ is a fracture below the stem level, and type Ⅳ is a fracture involving the tibial tuberosity. These four types are further classified into subtype A, indicating a stable tibial implant; subtype B, indicating a loose implant; and subtype C, for intraoperative fractures.

Management options depend primarily on two key factors: the fracture’s location and the tibial component’s stability, as described by the Mayo Classification system [15]. They can be broadly divided into conservative (non-operative) management, fracture fixation, and revision TKA (isolated tibial component or all the components) [1,4,5,10].

We present a case of early postoperative atraumatic lateral tibial plateau insufficiency PPF after primary TKA, which was treated successfully by revising the tibial component and reconstructing the lateral tibial plateau. 

## 2. Detailed Case Presentation

### 2.1. History

A female patient, 79 years old, presented with bilateral knee pain (not improving with analgesics), crepitations while bending her knees, and difficulty during stair climbing, and was diagnosed as having bilateral primary knee osteoarthritis (OA) with bilateral varus deformity. She had no significant comorbidities, apart from controlled hypertension; had no history of previous fractures; and had a BMI of 30.8 kg/m^2^ (Obesity Class I).

**Figure 1:**
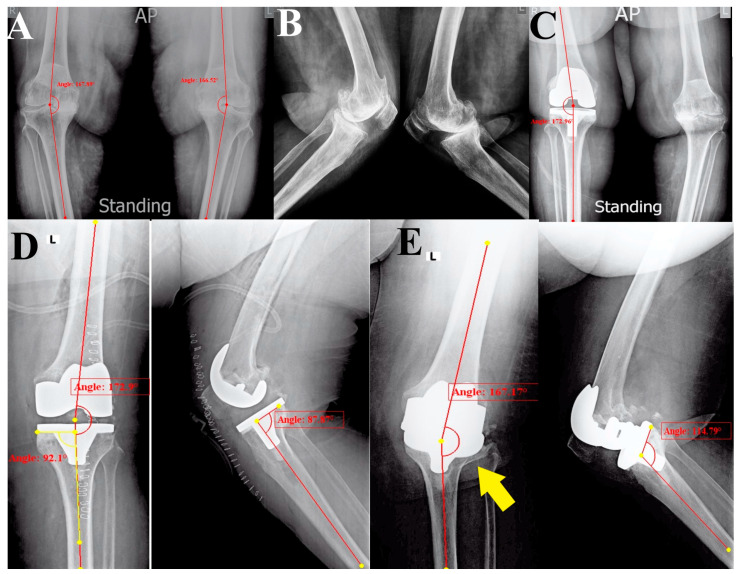
A series of initial radiographic evaluations (anteroposterior (AP) and lateral views); (**A**,**B**) the initial presentation had been more than four years ago with bilateral tricompartmental knee advanced osteoarthritis associated with varus deformity. (**C**) After having right knee TKA using a posterior stabilized prosthesis (four years ago). (**D**) Immediate postoperative radiographs after left TKA showed acceptable limb and individual implant alignment and positioning. (**E**) Three weeks postoperatively, catastrophic failure of the tibial component with lateral tibial plateau fracture (yellow arrow) and loosening of the tibial component.

She had had her right side replaced using a posterior stabilized (PS) TKA implant without a stem four years ago, and the follow-up of her right TKA was uneventful, but she still complained of left knee pain. In September 2023, she presented for left-side TKA. Her preoperative radiographic evaluation showed well-positioned right TKA and left knee advanced OA with varus deformity (anatomical femorotibial angle (aFTA) of the varus was 21°) (Figure 1A–C). 

The surgery was performed using a medial parapatellar approach under spinal anesthesia and tourniquet control. We used a cemented PS TKA implant without stems; the intraoperative course and the early postoperative period were uneventful. Immediate postoperative radiographs showed aFTA of valgus 7°, posterior tibial slope (TS) of 2°, and medial proximal tibial angle (MPTA) of 92°, indicating proper limb alignment and implant positioning (Figure 1D) [16]. 

Postoperatively, antibiotic prophylaxis (first-generation cephalosporin one gram IV) was continued for the first 24 h. Mechanical and chemical thromboprophylaxis was also used (subcutaneous low molecular weight heparin, 4000 IU, started 12 h postoperatively, then every 24 h till discharge). From the first postoperative day and under the supervision of a physiotherapist, functional training, including quadriceps strengthening, active knee motion, and walking (with support), was carried out.

She was discharged home two days postoperatively, and physiotherapy was started under the supervision of a physiotherapist. At a two-week follow-up, the staples were removed, no surgical wound problems were identified, and the patient reported good pain toleration and knee motion (active knee motion was from 0° to 90°). The patients presented three weeks postoperatively with a history of sudden atraumatic acute severe localized left knee pain, with an inability to bear weight on the left lower limb and a sense of knee instability.

### 2.2. Evaluation

Clinical: On the second day of the previously reported incident, she presented to our clinic in a wheelchair. She denied any history of trauma, fever, or wound discharge. On examination, she could not stand on the left lower limb; the left knee showed gross valgus deformity and was moderately swollen and grossly unstable, especially in the mediolateral directions. Maximum tenderness was localized over the lateral knee aspect, and the patella position was apparently normal compared to the contralateral side.

Laboratory investigation: The whole blood picture with differential WBC count was normal; however, ESR and CRP were slightly elevated.

Radiological: Plain radiographs (anteroposterior (AP) and lateral views) showed a valgus knee deformity (aFTA of valgus 13°) and PPF of the lateral tibial plateau with a loose tibial implant (classified as type ⅠB, according to Mayo Classification System [15]), as well as reversed TS of 25° (Figure 1E). 

### 2.3. Surgical Intervention

**Figure 2:**
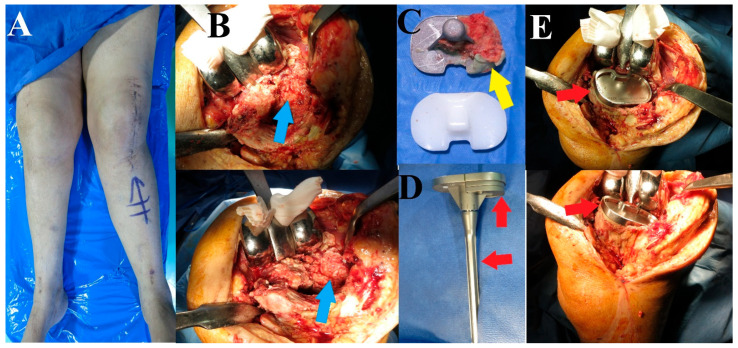
Intraoperative demonstration. (**A**) The gross valgus alignment of the left lower limb. (**B**) The knee was approached using the same approach as for the primary TKA (medial parapatellar); the femoral component was retained, and after removing the tibial implant, the defect of the lateral tibial plateau was evident (blue arrows). (**C**) The removed tibial implant showed bone attached to the lateral side (yellow arrow), indicating the fractured lateral tibial plateau. (**D**) The revision stemmed the tibial implant with two wedges (red arrows) to reconstruct the lateral tibial plateau bone defect. (**E**) The lateral tibial plateau cortex fracture was ignored (as it was inaccessible through the approach), and the final tibial component was implanted.

As the clinical picture did not suggest any signs of infection, we decided to manage the patient by revising the TKA (as the fracture was not amenable for fixation and was associated with a loose tibial component), where only the tibial implant would be revised. Examination under anesthesia confirmed the amount of knee instability (Figure 2A). The knee was approached using the same surgical approach as index surgery (medial parapatellar), with careful soft tissue handling. There was a moderate hematoma when opening the knee joint capsule, and the tibial plate was unstable (synovial fluid samples and five tissue samples were obtained from around the tibial component to exclude infection). The femoral component was stable and was protected against scratches throughout the surgery. We carefully tried removing the tibial plate by working on the bone–cement and cement–implant interfaces, especially on the medial side, to avoid further bone loss. After the removal of the tibial implant, the bone defect on the lateral side was evident (Figure 2B), and the fracture of the lateral tibial cortex could be felt, but was not accessible through the approach. The amount of bone loss could be appreciated from the cancellous bone attached to the lateral side of the removed tibial baseplate undersurface (Figure 2C). Reconstruction was performed using a stemmed tibial baseplate and two metal wedges to compensate for the lateral tibial defect (Figure 2D,E). The lateral tibial cortex fracture was ignored.

### 2.4. Postoperative and Follow-Up Protocol

The postoperative protocol was the same, and the patient was allowed to start immediate partial weight bearing (with support) and knee mobilization under the supervision of a physiotherapist. The obtained intraoperative tissue samples showed no bacterial growth, and the synovial WBC count was within the normal range (<200 cells/µL). The patient denied being screened for osteoporosis or having vitamin D or calcium supplements (which we prescribed for her); we advised her to consult an endocrinologist for osteoporosis assessment and management.

**Figure 3:**
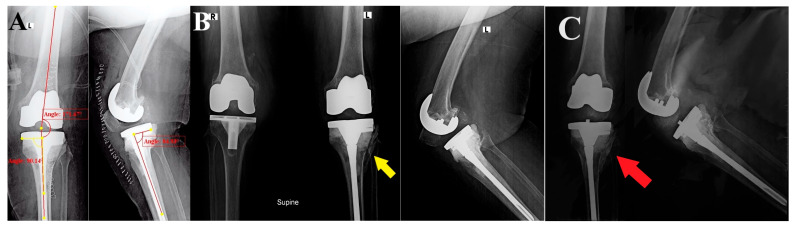
Postoperative and follow-up radiographs: (**A**) Immediate AP and lateral views of the revised TKA show the reconstruction of the lateral tibial defect, the restoration of the lower limb alignment, and proper implant positioning. (**B**) At eight weeks of follow-up, the limb and implant alignment was maintained, and the lateral tibial plateau cortex fracture showed signs of healing and callus formation (yellow arrow). (**C**) At six months of follow-up, the fracture showed healing (red arrow) and stable implants.

At two weeks, the staples were removed, and the surgical wound showed no signs of infection. After eight weeks of follow-up, the knee range of motion was from 0° to 100°, she reported satisfactory knee function according to Oxford Knee score (41 points), the radiographic assessment of the limb alignment and implant positioning was acceptable (aFTA: valgus 8°, posterior TS: 5°, and MPTA: 90°), and the lateral tibial plateau cortex fracture showed signs of healing and callus formation. The patient was unable to present at the clinic for further follow-up; upon contacting her (the last follow-up was at six months postoperative), she reported no advert incidents. The PROM outcomes in the form of Knee Injury and Osteoarthritis Outcome Score (KOOS) were assessed and a score of 72.6 was achieved, and she sent her last follow-up radiograph (Figure 3).

## 3. Discussion

The overall incidence of periprosthetic fracture after primary TKA was reported to reach up to 5.5% [17]. Although some authors have suggested a lower incidence of tibial PPF after primary TKA compared to the femoral side [11], the rates are relatively similar to those reported in the literature, ranging from 0.3% to 2.5% versus 0.4% to 1.7% for the femoral and tibial sides, respectively [17]. These fractures could be more common in patients with osteoporosis, female gender, inflammatory arthropathy, and obesity, as well as during revision surgery [14,18].

The current report describes a rare case of atraumatic type ⅠB PPF affecting the lateral tibial plateau in a relatively healthy female patient, which was treated successfully by revising the tibial implant and reconstructing the resultant bone defect using metal wedges.

The incidence of each fracture type or subtype varies among studies; in the report by Felix et al., the authors included 102 tibial PPFs; the most commonly occurring was type Ⅰ (59.8%); furthermore, 82% of these were subtype B (loose tibial baseplate). The authors reported that 90.1% of type Ⅰ fractures occurred in the medial tibial plateau, indicating the rarity of lateral tibial plateau fractures [15].

Femoral condyles insufficiency fractures post-TKA are well documented in the literature [8,9,19,20]. Although various systematic review articles have investigated tibial periprosthetic fractures after primary and revision TKA, to the best of our knowledge, insufficiency fractures have been less discussed [5,21]. In a recent systematic review by Abu-Mukh et al. investigating reports on tibial periprosthetic fractures over the past 30 years, they collected data on 287 patients from 23 studies. As a result, 78.8% of these fractures were due to either no history of trauma or low-energy trauma, but defining atraumatic fractures such as “insufficiency” were not reported [21].

In a systematic review by Ebraheim et al., the authors investigated 144 tibial PPFs after TKA; the most commonly reported fracture was type Ⅰ (55.36%), followed by types Ⅱ, Ⅲ, and Ⅳ, representing (21.4%), (21.4%), and (1.8%), respectively. Furthermore, subtype B was the most commonly occurring (43.75%), followed by types C and A, with percentages of 37.5% and 18.75%, respectively [4].

Although Ebraheim et al. reported that 78% of type Ⅰ fractures were atraumatic, the authors did not further differentiate these fractures into medial or lateral sides, especially as they included only two articles reporting on this particular fracture type. The first was a case report by Fonseca et al. where the fracture occurred on the medial tibial plateau after two years of having her index TKA; furthermore, the authors indicated that their patient had a fracture of the tibial stem, which could have caused a predisposition to varus tibial implant collapse and fracture [22]. The second study was conducted by Felix et al.; although the authors reported that 60.7% of type Ⅰ fractures were atraumatic, they did not mention how many medial or lateral sides fell within this atraumatic group [15]. What was mentioned previously supports the rarity of lateral tibial plateau atraumatic insufficiency fractures, as we have reported in the current case.

**Figure 4:**
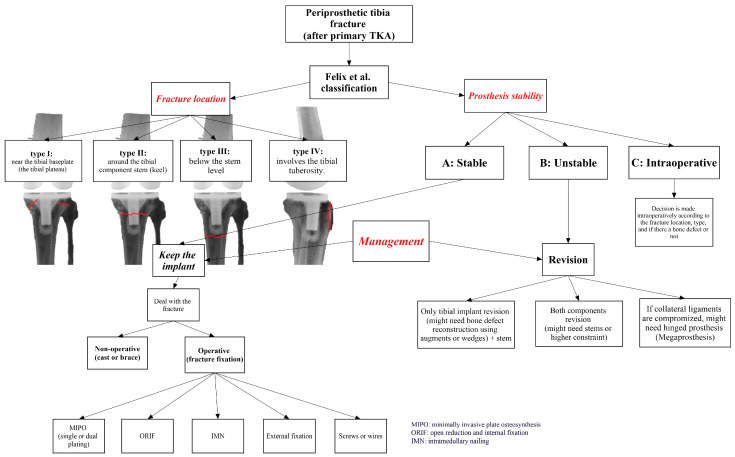
Various management options are based on the classification of Felix et al. [15].

Various management options were described to manage tibial PPFs after TKA (Figure 4), including non-operative methods (cast or brace), fracture fixation (using plates and screws or intramedullary nails or external fixators), revision TKA (single component or whole revision), and up to knee arthrodesis or amputation (if any other solution failed). These options rely mainly on the location of the fracture (type Ⅰ, Ⅱ, Ⅲ, and Ⅳ), the stability of the tibial component (subtype A or B), and the time of occurrence (subtype C, referring mainly to an intraoperative fracture) [11,15,18,21].

Each of the management techniques has its pros and cons. Although conservative management lines are simple, quick, and cheap, they carry a risk of fracture non-union, delayed mobilization, and the possibility of developing deep venous thrombosis. Fracture fixation provides better fracture stability with higher union rates, allowing for early rehabilitation at the expense of jeopardizing the soft tissue around the knee, in addition to the possibility of infection and sometimes difficult fixation if the proximal tibial bone stock is deficient. In situations where the implants are loose, revision surgery could provide immediate weight bearing, better rehabilitation, and acceptable functional outcomes, with the burden of increased surgical complexity and the added expenses [5,10,11,17].

In the current report, the fracture affected the lateral tibial plateau, leading to an obvious deformity and loosening of the tibial component, so the appropriate decision was to revise the tibial component. We reconstructed the lateral tibial bone defect by using two metal wedges, adding a stem to offload the tibial plateau area, and transferring the weight to the rest of the tibia.

Although we could not define the leading cause behind the PPF incident in the current case, we assumed it to be an insufficiency fracture considering the patient’s age, BMI, gender, and the atraumatic nature of the fracture. These factors have been reported in the literature to cause predisposition to insufficiency and atraumatic PPF around primary TKA, both on the femoral and tibial sides, as reported by Hoffmann et al., who described 35 out of 36 PPFs after primary TKA with an average BMI of 32.4 kg/m^2^ [23]. Moreover, female patients and those of advanced age were considered at higher risk, according to Singh et al. and Meek et al. [12,13].

Furthermore, some theories have been suggested in the literature regarding the occurrence of insufficiency PPFs after primary TKA, which could be related to the weakness of the unloaded compartment (lateral compartment in varus knee deformity) [9,24]. This possibility led to the idea of routine preoperative osteomalacia and osteoporosis investigation and optimization [6,7]; however, this is not routine in our institution, so in the absence of any history of previous fracture, we did not investigate the presence of osteoporosis or osteomalacia, which, honestly, we consider as a shortage from our side.

A second possibility is the occurrence of a nondisplaced fissure fracture of the tibial plateau while hammering the tibial implant in place, which propagates to a fully displaced fracture after the patient puts more weight on the knee, especially in obese patients. To overcome this possibility, some authors suggested adding a tibial stem if the surgeon is faced with obese patients and osteoporotic bone [25].

Lastly, component malpositioning (in varus–valgus direction or with malrotation) could lead to asymmetric load distribution, predisposing the patient to fatigue fractures of the proximal tibia [22].

We admit that the current report’s significant limitations are the lack of long-term follow-up and the inability to perform a bone quality assessment.

## 4. Conclusions

Early atraumatic insufficiency tibial plateau periprosthetic fracture is a rare issue that could complicate primary total knee arthroplasty. If the tibial component became loose, revision using a stemmed tibial component and reconstructing the tibial defect using metal augments or wedges is the management option of choice. We believe that in obese patients or if the bone quality is suspiciously poor, adding a tibial stem extension could help offload the tibial surface and guard against such early insufficiency fractures.

## Data Availability

The original contributions presented in the study are included in the article material, further inquiries can be directed to the corresponding author.
